# Review of the literature examining the correlation among DNA microarray technologies

**DOI:** 10.1002/em.20290

**Published:** 2007-06

**Authors:** Carole L Yauk, M Lynn Berndt

**Affiliations:** Environmental and Occupational Toxicology Division, Safe Environments Programme, Health CanadaOttawa, Ontario, Canada K1A 0K9

**Keywords:** microarrays, toxicogenomics, gene expression

## Abstract

DNA microarray technologies are used in a variety of biological disciplines. The diversity of platforms and analytical methods employed has raised concerns over the reliability, reproducibility and correlation of data produced across the different approaches. Initial investigations (years 2000–2003) found discrepancies in the gene expression measures produced by different microarray technologies. Increasing knowledge and control of the factors that result in poor correlation among the technologies has led to much higher levels of correlation among more recent publications (years 2004 to present). Here, we review the studies examining the correlation among microarray technologies. We find that with improvements in the technology (optimization and standardization of methods, including data analysis) and annotation, analysis across platforms yields highly correlated and reproducible results. We suggest several key factors that should be controlled in comparing across technologies, and are good microarray practice in general. Environ. Mol. Mutagen. 48:380–394, 2007. © 2007 Wiley-Liss, Inc.

## INTRODUCTION

**Fig. 1:**
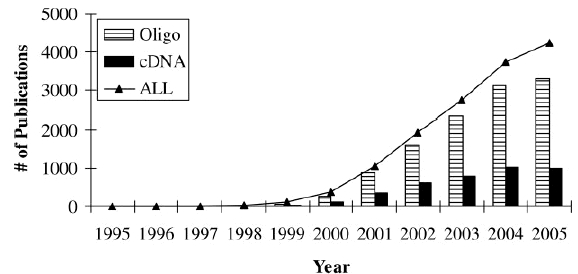
Number of publications retrieved from PubMed* using DNA microarray technologies. *PubMed search criteria: “microarray” [all fields] OR “microarrays” [all fields] OR “genechip” [all fields] OR “genechips” [all fields] AND “dna” [all fields] OR “cdna” [all fields] OR “complimentary dna” [all fields] OR “oligonucleotides” [all fields] OR “oligonucleotide” [all fields] Limits: XXXX [Publication Date].

DNA microarrays are quickly becoming standard tools in molecular biology, providing a powerful approach for the analysis of global transcriptional response. Over the past decade, microarrays have been widely used across biological disciplines and the number of published studies using the technology is still increasing (Fig. 1). As a result, the number of commercial suppliers of microarrays, associated reagents, hardware, and software continues to grow [Kawasaki, 2006; Technology Feature, 2006].

The diversity of microarray technologies and methods of data analysis have resulted in growing concern over the relationship among data obtained and published using different approaches. A number of impressive efforts have recently been made to develop standards for microarray experiments including the Minimum Information About a Microarray Experiment (MIAME) guidelines [Brazma et al., 2001; http://www.mged.org/Workgroups/MIAME/miame.html], the External RNA Controls Consortium (ERCC) [Baker et al., 2005; http://www.cstl.nist.gov/biotech/Cell&TissueMeasurements/GeneExpression/ERCC.htm], and the Microarray Quality Control project (MAQC) [http://www.fda.gov/nctr/science/centers/toxicoinformatics/maqc/; Shi et al., 2006]. These projects have made significant advances toward improving the evaluation of microarray data quality and the reproducibility of results among laboratories and platforms. A large majority of journals have made submission of microarray data to publiclyavailable repositories and adherence to the MIAME standards compulsory for publication of experiments utilizing DNA microarrays. Adherence to established standards, alongside proven reproducibility and correlation within and between datasets produced by different microarray platforms, is essential for the usefulness of such databases. Furthermore, establishing the correlation and reproducibility among different microarray technologies is important for the validation of microarrays as robust, sensitive, and accurate detectors of differential gene expression.

Over 40 studies have been carried out since 2000 to evaluate the extent to which data produced by different microarray technologies correlate. In this review, we summarize the cross-platform studies designed to examine the correlation of gene expression profiles and differentially expressed genes among different DNA microarray technologies. The potential reasons for discrepancies reported in earlier comparative studies, and the methodological changes which led to improved correlations generally reported in more recent publications are discussed.

## A BRIEFOVERVIEWOF TECHNOLOGICAL AND ANALYTICAL CHOICES

**Fig. 2:**
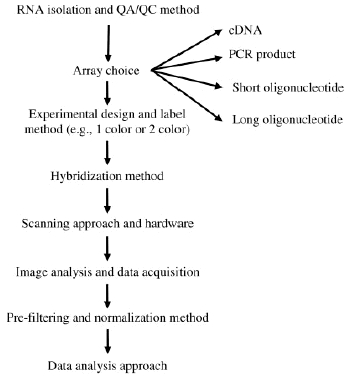
Summary of choices for microarray experiments.

Various technical and analytical options are available for microarray experiments (Fig. 2). These options are sometimes governed by the selection of microarray platform. For example, a decision to use Affymetrix chips [http://www.affymetrix.com/; McGall et al., 1996] limits the choice of scanner, and subsequent steps through to image analysis, to those supplied by Affymetrix. The Affymetrix technology uses a combination of oligonucleotide synthesis and photolithography to position specific oligonucleotide probes in a predetermined spatial orientation. Each gene is represented by a series of different oligonucleotide probes spanning the coding region of that gene [Liu et al., 2003]. Each oligonucleotide probe is paired with a mismatch probe in which the central base in the sequence has been changed. Therefore, application of the Affymetrix system is heavily directed by the manufacturer's recommendations. However, for experiments using microarrays that are spotted on glass microscope slides, a number of alternatives are available that may contribute to variation in the data acquired (Fig. 2).

Several comprehensive reviews cover different microarray platforms and approaches [Sevenet and Cussenot, 2003; Hardiman, 2004; Stoughton, 2005; Ahmed, 2006a,b; Kawasaki, 2006]; readers are directed to these sources for a more in-depth overview of microarray technologies. Below, some typical options that can contribute to technical variation in the gene expression measurements acquired between different technologies are briefly summarized (Fig. 2).

Probe choices for microarrays may include amplified cDNA clones, PCR gene products, or different lengths of oligonucleotides [Kawasaki, 2006]. Studies examining the correlation among microarray technologies have focused primarily on differences between probe types. However, many other factors contribute to technical variability. Methods of printing/deposition of probes onto glass slides include contact-spotting using pins, deposition by ink jet, or in situ synthesis of oligonucleotides on the slide [Hughes et al., 2001; Gao et al., 2004]. Slide surfaces may be coated with different types of matrices that govern the affinity of probe binding and affect background fluorescence [Rickman et al., 2003; Sobek et al., 2006]. Target preparation varies and may include different amounts of starting RNA, amplification, and labeling methods [Gold et al., 2004; Hardiman, 2004; Schindler et al., 2005; Singh et al., 2005; Kawasaki, 2006], all of which contribute to the type and quality of data produced. In addition, cDNA and several oligonucleotide platforms allow experiments to be carried out in one or two colors [Patterson et al., 2006]. Two color experiments may involve dye-swap, reference RNA, or loop designs [Vinciotti et al., 2005; Patterson et al., 2006]. Hybridization can be undertaken manually or using automated hybridization stations; optimization of methods is important to minimize array variability and hybridization artifacts [Yauk et al., 2005; Han et al., 2006; Yauk et al., 2006]. The scanner (high or low laser powers) and scanner settings influence background fluorescence, the number of saturated spots and the number of spots below background [Shi et al., 2005b; Timlin, 2006], and should be adjusted to maximize the linear dynamic range. Acquisition of data from images can be carried out using various algorithms through different commercial packages. The final critical steps include applying the appropriate filtering methods, evaluating microarray data quality [Shi et al., 2004], normalization [Bilban et al., 2002b; Quackenbush, 2002], and data analysis [Shi et al., 2005a; Jeffery et al., 2006]. Normalization and detection of differential gene expression are key to ensuring the accuracy and reproducibility of data across time, laboratories, and platforms, and are reviewed in detail elsewhere [Bilban et al., 2002b; Quackenbush, 2002; Armstrong and van de Wiel, 2004; Reimers, 2005; Breitling, 2006].

## CORRELATION AMONG TECHNOLOGIES

Data obtained from different commercially-made and in-house microarray platforms have been compared in a large number of studies. Experiments have been carried out to determine the effective differences in accuracy (proximity to true value), sensitivity (ability to accurately detect changes at low concentrations), and specificity (to hybridize to the correct gene) among the technologies [Hardiman, 2004; van Bakel and Holstege, 2004; Draghici et al., 2006]. Intra-platform variability and reproducibility have been used as measures of the quality of the data produced for individual platforms. Several of these studies have been aimed at answering the question “which platform is the best?” The answer to this question is inarguably experiment-specific. A more relevant question is ‘which platforms generate comparable and reproducible data?’.

In the remaining sections, experiments investigating the reproducibility of data among DNA microarray technologies and the correlation among these data with respect to expression profiles and the identification of differentially expressed genes are reviewed.

## EARLY STUDIES: 2000–2003

For the sake of simplicity, the discussion has been separated into early (years 2000–2003) and later (years 2004 to present) studies. Comparative studies began to change around 2004 when investigators began to: apply larger sample sizes, include more microarray platforms, examine relationships among laboratories, employ more sophisticated bioinformatics approaches, and generally find datasets to be more correlated. In addition to being limited in scope (i.e. comparing only 2–3 technologies or using small sample sizes), the early studies focused heavily on the comparison of cDNA microarrays to other technologies.

Table I summarizes all of the experiments that we identified that examined cross-platform performance prior to 2004. Of 13 studies, 8 produced results supporting the reproducibility and concordance of data across different microarray technologies. These findings should be interpreted with caution, as they are based on the authors' conclusions, and the term “agree” is somewhat ambiguously defined. At a time when expectations for the prospects of DNA microarrays were extremely high, these negative findings were discouraging. For example, a widely cited article by Tan et al. [2003] examined gene expression of technical and biological replicates of RNA on Codelink (Amersham oligonucleotide), Affymetrix, and Agilent cDNA arrays. Although internal consistency was high, Pearson correlation coefficients were moderate to poor across technologies (0.48–0.59). Similarly, a comprehensive investigation of 56 NCI-cell lines using 2 array technologies (Affymetrix and cDNA) showed poor correlations between the datasets [Kuo et al., 2002]. These findings led researcher to conclude that the general outlook for comparing across laboratories and platforms was bleak [Hardiman, 2004]. It was hypothesized that the discrepancies in these studies arose from intrinsic differences in the properties of the arrays, as well as the processing and analysis of the data. As a result, it was suggested that data from microarray analyses be interpreted with caution.

**TABLE 1:** Studies Examining the Correlation Among Microarray Technologies from 2000 to 2003

Publication	Platforms	Probe ID	Validation	Author'conclusion
Kane et al. [2000]	Operson 50mer, PCR probes	Sequence similarity	None	Agreement
Huges et al. [2001]	Agilent oligo, cDNA	Sequence similarity; not clearly specified	None	Agreement
Yuen et al. [;2002]	Affymetrix, custom cDNA	Sequence similarity (47 genes studied, all sequence confirmed on cDNA platform)	Quantitative (Q) RT-PCR	Agreement
Kuo et al. [2002]	Affymetrix, cDNA	Sequence similarity: For each cDNA probe a best matching probe set was identified by sequence alignment.	None	Disagreement
				Poor correlation between technologies. 56 cell lines from the NCI-60 cell lines studied; independent microarray experiments compared from two labs using different materials and protocols. Cells were cultured independently and all were processed separately. Observed that cross-hybridization of genes to various probes reduced correlation of cDNA arrays to the oligonucleotide arrays. Low abundant transcripts performed poorly. Subsequent re-analysis and improvement by sequence matching [Carter et al., 2005]
Kothapalli et al. [2002]	Affymetrix, Incyte cDNA	Sequence similarity: A subset of clones were sequence verified	Northern	Disagreement Noted that a large proportion of cDNAs were incorrectly annotated. Discrepancies were related to probes
Li et al. [2002]	Affymetrix, Incyte cDNA	Unigene or Genbank	QR-T-PCR	Disagreement Conclusion based primarily on sensitivity and specificity–Affymetrix found 218 genes differentially expressed versus 4 for cDNA
Barezak et al. [2003]	Affymetrix, Operon 70mer	Unigene ID	None	Agreement
Carter et al. [2003]	Agilent oligonucleotide, cDNA	Sequence matched	QRT-PCR	Agreement
Lee et al. [2003]	Affymetrix, cDNA	UniGene	QRT-PCR	Agreement
Wang et al. [2003]	cDNA, Custom oligo	Sequence similarity	RT-PCR	Agreement
Rogojina et al. [2003]	Affymetrix, Clontech cDNA	Genbank ID	QRT-PCR and Q-immunoblot	Disagreement Small sample size; examined list of genes that arbitrarily had 1.7-fold or greater change
Tan et al. [2003]	Affymetrix, Agilent cDNA, Amersham 30mer	Genbank ID	None	Disagreement Correlations in expression level and significant gene expression were divergent. Subsequent reanalysis and improvement by Shi et al. [2005a] (see Table II)
Baum et al. [2003]	Febiot Geniom, Affymetrix, cDNA	Not described	QRT-PCR	Agreement

**TABLE II:** Studies Examining the Correlation Among Microarray Technologies from 2004 to 2006

Publication	Platforms	Probe ID	Validation	AUthors' conclusion
Yauk et al. [2004]	Affymetrix, Agilent cDNA, Agilent oligo, Codelink (Amersham), Mergen, NIA cDNA	Unigene ID	None	Agreement Good platforms correlate well. Expression profiles clustered by biology rather than technology. cDNA platforms less sensitive than oligonucleotide
Woo et al. [2004]	Affymetrix, in-house spotted cDNA and oligonucleotide	MGI identifiers	None	Agreement Good concordance in expression level and statistical significance between Affymetrix and oligonucleotide arrays; cDNA arrays showed poor concordance with other platforms
Jurata et al. [2004]	Affymetrix, Agilent cDNA	Unigene ID	Real time RT-PCR	Moderate agreement Gene changes overlapping between the two platforms were co-directional; RT-PCR validation rates were similar
Park et al[2004]	Affymetrix, Agilent cDNA, custom oligonucleotides from three different sources printed by Agilent	Unigene ID	Real time RT-PCR	Agreement Expression level correlation low, but log ratios high; correlation is stronger for highly expressed genes
Mecham et al. [2004a]	Affymetrix, Agilent cDNA	Sequence matched	None	Agreement Cross-platform analysis greatly improved by sequence matching
Mah et al. [2004]; Two different labs	Affymetrix, cDNA	Unigene (sequence matched)	Real time RT-PCR	Disagreement Poor correlation when matched by expression level
Jarvinen et al. [2004]	Affymetrix, Agilent cDNA, custom-cDNA	Unigene ID	None	Moderate agreement Good correlations for commercial, whereas the correlations between the custom-made and either commercial platforms were lower. Discrepant findings due to clone errors, old annotations, or unknown causes
Shippy et al. [2004]	Affymetrix, Codelink (Amersham)	Unigene ID	Real time RT-Pcr	Agreement After noise adjustment (use precent)genes
Ulrich et al. [2004];ILSI/HESI collaboration	Affymetrix, Clontech, Incyte, NIEHS, Molecular Dynamics, PHASE-1	Comparison of pathways	Real time RT-PCR	Agreement Correlation of the biological pathways involved in response to toxicant exposure
Stec et al. [2005]	Affymetrix, Millennium Pharmaceuticals cDNA	UniGene ID, then sequence matched using BLAST	None	Moderate agreement Increased significantly after sequence matching; discrepant correlations between Affymetrix and cDNA measurements could be explained by probe sequence differences
Irizarry et al. [2005]; Lab-lab comparison (10 labs)	Affymetrix (5 labs), cDNA (3 labs), 2-color oligonucleotide (Qiagen 70mer; analyzed in 2 labs)	Unigene, locuslink, RefSeq	Real time RT-PCR	Agreement Among best performing laboratories; increased data quality with more stringent pre-processing
Dobbin et al. [2005]; Cross-laboratory comparison	Affymetrix (inter-and intra-laboratory correlation)	None required	None	Agreement Intra-laboratory correlation was only slightly stronger the inter-laboratory. Samples clustered by biology rather than laboratory
Larkin et al. [2005]	Affymetrix, TIGR cDNA	Sequence mapped TIGR	Real time RT-PCR	Agreement Biological treatment had a greater effect on gene expression than platform for 90% of the genes
Bammler et al. [2005]; Lab-lab comparison 7 labs, 12 platforms	Affymetrix, Agilent Amersham (Codelink), Compugen, Operon, 2 custom oligo, 5 custom cDNA,	Transcripts matched using NIA mouse index	None	Moderate agreement Standardized protocols and data analysis required
Pylatuik and Fobert [2005]	Affymetrix, Genomic Amplicon arrays, Operon oligo	Locus ID (Arabidopsis)	Northern blot	Moderate agreement Signal intensity-dependant
Shi et al. [2005a]	Tan et al., 2003; dataset	Genbank acc.No	N/A	Agreement Concluded that the quality of the original dataset was poor and inappropriate methods were applied for analysis. Alternate analysis had 10× >concordance
Bames et al. [2005]	Affymetrix, Illumina Beadarrays	Sequence matched using BLAST [Kent, 2002]	None	AgreementFor genes with high expression and concordance improved for probes that were verified to target same transcript
Gwinn et al [2005]	Affymetrix, Amersham (Codelink), cDNA	Amersham (locuslink ID) Affymetrix (GenBank)–links made via IDs given by company; genes of interest used probe sequence	Real-time RT-PCR	Disagreement Each platform yielded unique gene expression profiles
Petersen et al. [2005];	Affymetrix, in-house cDNA, in-house Operon oligonucleotide	Unigene	Real time RT-PCR	Agreement High concordance for significant expression ratios and 1.5 to 2-fold changes (93–99%)
Carter et al. [2005]	Affymetrix, Stanford cDNA	Sequence matched	None	Agreement Re-examination of NCI-60 cell line data. Overlapping probes correlate well
Wang et al. [2005]; 5 data sets were either collected from a public source or generated in house	Affymetrix, Agilent oligo, cDNA	Unigene ID	None	Agreement Data were more consistent between two commercial platforms and less consistent between custom arrays and commercial arrays; expression at the gene level exhibited an acceptable level of agreement. Lab and sample effect was greater than platform effect
Schlingemann et al. [2005]	Affymetrix, in-house long oligo	Unigene ID	Real time RT-PCR	Agreement Similar profiles and strong correlations were found for the 2 platforms
Warnat et al. [2005]	6 different cDNA and oligo array studies previously published from several laboratories	Unigene ID	None	Agreement Integrated raw microarray data from different studies for supervised classifications. More platforms better for predictive analysis
Ali-Seyed et al. [2006]	Affymetrix, Applied Biosystems	Promote analysis	Real time RT-PCR	Agreement AB more sensitive and more correlated with RT-PCR
Severgnini et al. [2006]	Affymetrix, Amersham (codelink)	Locuslink ID	Real time RT-PCR	Disagreement Only 9 genes found to be differentially expressed in common out of 42 (Affymetrix) and 105 (Codelink) in total
de Reynies et al. [2006]	Affymetrix, GE Healthcare (Amersham), Agilent	Sequence mapped	Real time RT-PCR	Moderate agreement 1 color more precise than 2 color; Affymetrix and Agilent were more concordant based on detection of differential genes
Wang et al. [2006]	Agilent, Applied biosystems	Sequence matched (BLAST)	Real time RT-PCR	Agreement 1375 genes confirmed with RT-PCR
Kuo et al. [2006]; Lab-lab comparison as well	Affymetrix, Amersham, Mergen, ABI, custom cDNA, MGH, MWG, Agilent, Compugen, Operon	Probes sequence matched within 1 exon (Unigene, LocusLink, RefSeq, Refseq exon)	Real time RT-PCR	Strong agreement Commercial better than in-house, 1-color better than 2-color
Shi et al. [2006]; Lab to lab comparison (3 sites); used commercial RNA sources; MAQC	Affymetrix, Agilent (1 and 2 color), Applied Biosystems, Eppendorf, GE Healthcare, Illumina, in-house spotted Operon oligonucleotide	Probes sequence mapped to RefSeq and to AceView using 30 probe for genes with multiple oligonucleotides	Real time RT-PCR; TaqMan1 (Roche Molecular Systems); StaRTPCR and QuantiGene carried out by Canales et al. [2006]	Strong agreement Intra-platform consistency across test sites and high inter-platform concordance with respect to differentially expressed genes; high correlation between QRT-PCR values and microarray results
Guo et al. [2006]; Included 2 test sites for Affymetrix; MAQC	Affymetrix, Agilent, Applied Biosystems, GE Healthcare	Probes sequence mapped to RefSeq	None	Agreement High inter-site and cross-platform concordance in the detection of differential gene expression using fold change rankings; fold change ranking outperforms other analysis methods
Canales et al. [2006]; MAQC	Affymetrix, Agilent, Applied Biosystems, Eppendorf, GE Healthcare, Illumina	Probes sequence mapped to RefSeq	Real time RT-PCR; TaqMan1 (997 genes), StaRT-PCR (205 genes), and QuantiGene (244 genes)	Agreement High correlation between gene expression values and microarray results. Main variable was probe sequence and target location

Subsequent studies revisited both of the datasets described earlier (an excellent demonstration of the utility of making microarray data publicly–available) and were able to reanalyze the data. Reanalysis revealed significantly improved correlations, providing insight into the basis for discrepancies found among the technologies. Shi et al. [2005a] examined the Tan et al. [2003] data in more detail and found that intra-platform consistency was generally low, suggesting that experimental protocols may not have been optimized for the array platform used. Furthermore, by applying more appropriate statistical tests (examining ratios instead of absolute measurements of gene expression) they were able to significantly increase the correlation coefficients obtained in comparisons of the technologies. Shi et al. [2005a] concluded that a combination of low intra-platform consistency and poor choice of data analysis procedures were the cause for discordance among the datasets, rather than inherent technical differences among the platforms as suggested by Tan et al. Data produced by microarray hybridization of RNA from NCI-60 cell lines [Kuo et al., 2002] were re-evaluated by stringent sequence mapping [Carter et al., 2005] of matched probes. By redefining probe sets, a substantially higher level of cross-platform consistency and correlation was found. The authors concluded that by using probes targeting overlapping transcript sequence regions a greater level of concordance can be obtained compared to using UniGene ID or other sequence-matching approaches. It should be noted that the study by Kuo et al. was carried out in two different laboratories using cells cultured independently, rather than using the same RNA samples matched for both platforms. Therefore, real biological variability will cause differences in the two datasets produced.

These early studies were key to identifying potential sources of discrepancies between microarray datasets, highlighting the need to investigate this issue in more detail. As microarray technologies, annotation, and techniques for analysis continue to be refined, a number of important sources of error and data misinterpretation have been identified in these early studies and are summarized in subsequent sections.

## LATER STUDIES: 2004 TO PRESENT

In 2003, discrepancies in the literature led us to carry out our own cross-platform evaluation [Yauk et al., 2004]. Gene expression from three replicates of three different RNA sources (mouse whole lung, mouse lung cell line, and Stratagene Universal mouse reference RNA) were evaluated with six different technologies encompassing different reporter systems (short oligonucleotides, long oligonucleotides, and cDNAs), labeling techniques, and hybridization protocols. We were unable to match probes through sequences, because not all platform providers made sequence information available (probes were matched by UniGene ID). By applying rigorous filtering and normalizations, and using an adequate sample size, we found that the top performing platforms exhibited low levels of technical variability which resulted in an increased ability to detect differential expression, and that biology, rather than technology, accounted for the majority of variation in the data when normalized ratios were examined. Subsequent studies have confirmed that with improved technologies, annotations, statistical rigor, and experimental design, the data from different microarray platforms are highly comparable.

Table II summarizes the studies identified that examined the correlation among microarray technologies within the last 3 years (2004 to present). Among the 32 studies that we identified, only three concluded that microarray platforms do not correlate well (<10%). Careful examination of these studies reveals some potential errors that the authors may have made in reaching these conclusions. The remaining studies show a moderate to high level of correlation among technologies.

### Studies With Poor Correlation

Mah et al. [2004] examined RNA expression profiles of human colonoscopy samples using [^33^P]dCTP labeling and hybridization to probes generated from a human cDNA clone set spotted on nylon filters, compared to data generated from human Affymetrix HG-U95Av2 chips. The authors found weak correlations using Spearman rank order coefficients on normalized signal intensities between the two systems for sequence-matched probes. Examination of absolute expression of genes fails to account for the major role that different probe sequences and location will play in resulting signal intensity. The same transcript can produce different signal intensities for different probes; even over-lapping probe sequences targeting the same transcript can produce different signal intensities [Draghici et al., 2006]. More recent studies emphasize that examination of log ratios rather than expression intensities will greatly increase the observed correlation coefficients [Park et al., 2004]. While analyses based on signal intensities are appropriate for case-control study designs using a single platform, the use of signal intensities is not appropriate for cross-platform comparisons. Therefore, the measurement of ratios may have yielded increased correlation in the Mah et al. analysis.

Severgnini et al. [2006] compared Amersham Codelink and Affymetrix microarrays through hybridization of four samples (Human breast cancer cell line MDA-MB-231; two treated and two untreated samples). The authors carried out a hierarchical cluster analysis on genes matched by LocusLink IDs on normalized expression levels and found poor correlation coefficients and clustering. Again, this negative result likely reflects analysis on normalized signal intensities rather than on ratios. Investigation of differentially expressed genes was carried out independently for both platforms (rather than on the genes in common only, filtered for poor/saturated/absent genes). The authors found 105 genes differentially expressed on Affymetrix, and 42 on Codelink, 9 of which were found in common. A more appropriate analysis would have been to examine differential expression on the filtered set of genes in common only. Therefore, a combination of inappropriate statistical analysis and small sample size may have contributed to the negative findings in this study.

Lastly, [Gwinn et al., 2005] found minimal similarity between Affymetrix, Amersham Codelink and NCI cDNA platforms when they analyzed three technical replicates of a human cell culture exposed to benzo[a]pyrene compared to three technical replicates of a control sample. We suggest a few potential reasons for low correlations found in this study. First, probes were generally matched using gene information provided by the manufacturer. In the latter part of the study, the authors re-examined their data and carried out some sequence investigation but the results were not fully presented and appear to be inconclusive. Other potential factors contributing to the poor correlation observed include: (a) normalizations and analyses were not carried out for all platforms combined, but were carried out individually within a platform; (b) differential expression was investigated using a small sample size (*n* = 3) for a subtle toxicological effect (e.g., Affymetrix only found 23 genes differentially expressed); (c) signal log ratios (SLR) were arbitrarily defined as differentially expressed if ±0.6 (with no measure of variability or statistic presented), while similarity to the other platform was arbitrarily defined as within ±0.2 SLR for the same gene on another platform; (d) it is unclear what level of filtering was applied to examine the correlation among the genes that were in common among the platforms.

### Studies With Moderate to High Correlation

Since 2004, the vast majority (29/32) of technical papers comparing microarray platforms have generated results that show a moderate to high degree of correlation among the technologies. Several of these studies have been very comprehensive encompassing many microarray platforms analyzed in both one and two colors, employing different probe types spotted both in-house and commercially, and using data from the same samples analyzed in several different laboratories. By fine-tuning approaches and analyses, these studies identified methods to yield increased correlation among laboratories and platforms. Below, we present general findings that have resulted in increasing our understanding of how microarray platforms relate to each other, and we discuss a few of the more comprehensive studies.

Several studies have demonstrated that using a sequence-driven rather than an annotation-driven approach to analyzing data from different platforms yields improved correlation among technologies [Mecham et al., 2004a; Carter et al., 2005; Kuo et al., 2006]. Annotation of microarray platforms has improved greatly over the past several years, but errors in annotation will continue to affect analyses until genomes are completely validated and curated. In addition to potentially matching incorrect probes due to errors in annotation, sequence-driven matching improves correlation by ensuring that probe pairs are examining similar gene regions. The re-examination of the NCI cancer cell lines [Carter et al., 2005] using sequence-driven probe matching, described earlier, exemplifies the importance of ensuring the appropriate comparisons of probes/genes are made in cross-platform analyses. Similarly, Stec et al. [2005] compared platforms using either UniGene identifiers or by sequence matching using BLAST alignments. They found higher correlations when the Affymetrix probe identifiers were sequencematched to ensure they fell within the cDNA probes. Mecham et al. [2004a] also found significantly improved correlation for Affymetrix compared to cDNA platforms using sequence matching. Sequence matching eliminates errors introduced by mis-annotation, and potential discrepancies introduced by probes aligning with multiple family members or alternative transcripts. Probes targeting regions of a gene in close proximity (e.g. within the same exon) are more likely to have highly correlated expression ratios [Canales et al., 2006; Kuo et al., 2006].

A number of the later studies highlight the importance of removing unreliable data from experiments prior to analysis, termed filtering. These studies generally found that probes for genes with strong expression signal tended to give more highly correlated results than those with weaker signals [Park et al., 2004; xShippy et al., 2004; Barnes et al., 2005; Pylatuik and Fobert, 2005; Kuo et al., 2006]. Signal within the background range is highly variable and contributes to much of the noise observed in microarray datasets [Bilban et al., 2002a; Park et al., 2004; Shippy et al., 2004; Draghici et al., 2006; Kuo et al., 2006]. Most commercially-available image-acquisition programs now have implemented algorithms to flag poor quality, low signal, and saturated spots. Filtering methods applied to microarray datasets prior to analysis increases the correlation among technologies [Pounds and Cheng, 2005; Kuo et al., 2006].

Optimization and standardization of protocols ensures that data produced within a technology is reproducible. Intra-platform reproducibility is obviously required before inter-platform relationships can be evaluated. A number of the later studies concluded that concordance was high among the best performing laboratories, platforms, or for commercial compared to in-house microarrays [Jarvinen et al., 2004; Yauk et al., 2004; Bammler et al., 2005; Irizarry et al., 2005; Wang et al., 2005; Kuo et al., 2006]. These results were likely due to the optimization of protocols within laboratories that routinely use a technology, technical expertise acquired in laboratories that use the platform routinely, and increased standardization through use and development of commercially-available microarrays compared to in-house microarrays. Improvements in methodology, the development of quality control standards and references, and the implementation of standards for data analysis will improve the relationship among data produced by different microarray platforms [Bammler et al., 2005].

Recently, a number of large-scale efforts have produced comprehensive studies evaluating microarray performance across technologies and laboratories. Members of the Toxicogenomics Research Consortium [Bammler et al., 2005] examined data produced by seven laboratories and 12 microarray platforms. Each laboratory was provided with aliquots of two different RNA samples (one liver RNA sample and one mixture of tissues). They found that correlation across platforms and laboratories was generally poor. However, by implementing standardized protocols for RNA labeling, hybridization, filtering, processing, data acquisition, and normalization, increased reproducibility was obtained. Unsurprisingly, raw intensity values correlated poorly. The highest levels of reproducibility obtained were between laboratories using commercial arrays and applying standardized protocols. This analysis yielded median correlation coefficients of 0.87–0.92. The consortium concluded that the microarray platform has a large effect on the variability in the data, and standardization is required to generate data that are reproducible across laboratories. However, the group also noted that reproducibility among platforms was generally very high when analyses were carried out on biological categories identified by gene ontology analysis.

Irizarry et al. [2005] examined microarray data produced by 10 different laboratories from three different platforms using the same RNA samples. Measurement of relative expression (e.g. ratios), rather than absolute measures of gene expression were found to correlate well among the best performing laboratories. The authors emphasize the importance of experience and expertise with a platform before a laboratory can produce accurate and reproducible data, and that laboratory effect can be a strong variable (>platform effect). Furthermore, the authors stress the importance of pre-processing (normalization) before making any cross-platform comparisons.

Kuo et al. [2006] examined correlation among five different platforms encompassing both cDNA and oligonucleotide microarrays, one and two-color hybridizations, commercial and in-house chips and including results generated in two different laboratories. They matched probes at the gene id, gene, and exon level using UniGene, LocusLink, RefSeq, and RefSeq exon. Data mapped through probe sequences were more correlated than through other identifiers. Log ratios showed high correlation (0.63–0.92) for all platforms except academic cDNA and Compugen. Spot quality filtering had a strong positive effect on correlation coefficients. Inter-laboratory Pearson and Spearman correlations for log2 ratios were high within platforms (0.79 for Mergen; 0.89 for Affymetrix; 0.93 for Amersham). Quantitative RT-PCR was carried out for 160 genes and agreed well with the microarray platforms, although RT-PCR had a larger dynamic range. The authors concluded that with stringent preprocessing and sequence matching, consistency and reproducibility among platforms and laboratories was good for highly expressed genes and variable for genes with lower expression.

A large scale real-time PCR validation experiment was conducted by Wang et al. [2006]. The authors used TaqMan® gene expression to evaluate the performance of Agilent and Applied Biosystems (AB) microarrays for 1375 genes. The authors compared log_2_ fold-changes and found that the dynamic range was greatest for RT-PCR, followed by AB and then Agilent. Despite differences in the dynamic range, moderate to strong correlations of fold change were found for AB (*R*2 = 0.71–0.75) and Agilent (0.45–0.52). The estimated range of fold changes (in log_2_ scale) was from −10 to 10 for TaqMan®, −4 to 6 for AB, and −2 to 2 for Agilent, indicating ratio compression for microarray platforms. Ratio compression was expected because of various technical limitations (e.g., narrower dynamic range, signal saturation, and cross-hybridizations). In the analysis of differential expression, the authors noted that sensitivity and specificity were highest for genes with high and medium expression levels, compared to those with low expression levels.

The MicroArray Quality Control (MAQC) project evaluated inter-and intra-platform reproducibility in a series of papers [Canales et al., 2006; Guo et al., 2006; Patterson et al., 2006; Shi et al., 2006; Shippy et al., 2006]. The project was led by US Food and Drug Administration scientists and involved 137 participants from 51 organizations. Shi et al. [2006] presented data evaluating five replicates of two distinct, high-quality RNA samples from four titration pools using seven microarray platforms (each platform was evaluated at three independent tests sites). Probe sequences were mapped to the RefSeq human mRNA database [http://www.ncbi.nlm.nih.gov/RefSeq/; Pruitt and Maglott, 2001; Pruitt et al., 2005] and to the AceView database [Thierry-Mieg and Thierry-Mieg, 2006]. The relative expression between matched probes was examined. Rank correlations of the log ratios were in good agreement between all sites, with a median of 0.87 (lowest was *R* = 0.69). Generally, differentially expressed genes showed an overlap of at least 60%, with many comparisons yielding 80% or more between platforms, and 90% within platforms (between sites). An average overlap of 89% was found between test sites using the same platform and 74% across one-color microarray platforms. The Affymetrix, Agilent, and Illumina platforms showed correlation values of 0.90 to TaqMan® assays, while GE Healthcare and NCI had an average of 0.84. The results were validated using two additional quantitative gene expression platforms [Canales et al., 2006] that also showed high concordance. In addition, toxicogenomics data generated from rats exposed to aristolochic acid, riddelliine, and comfrey and analyzed using four different microarray platforms were evaluated [Guo et al., 2006]. These data showed high concordance in inter-laboroatory and cross-platform comparisons. The results of the MAQC project provide strong support for inter-platform consistency and reproducibility and support the use of microarray platforms for the quantitative characterization of gene expression.

## SUMMARY OF FACTORS LEADING TO HIGHER CORRELATION AMONG TECHNOLOGIES

Later studies resolved many of the issues surrounding the lack of correlation found in earlier studies. Sources of error in the early cross-platform microarray experiments can be divided into problems resulting from the platform and protocols, and those that result from the experimental design or method of analysis.

## Platform Issues

One of the most important problems that arose in early studies was incorrect annotation of probes on the various microarray platforms. For many cDNA platforms, sequencing of clones from the libraries spotted revealed that a large number were incorrect or contaminated [Halgren et al., 2001; Taylor et al., 2001; Kothapalli et al., 2002; Jarvinen et al., 2004; Kuo et al., 2006]. Errors in annotation were not exclusive to cDNA platforms. For example, Mecham et al. [2004b] examined mammalian Affymetrix microarrays and found that greater than 19% of the probes on each platform did not correspond to their appropriate mRNA reference sequence. Dai et al. [2005] investigated Affymetrix probe information and concluded that the original probe set defi-nitions were inaccurate, and many previous conclusions derived from GeneChip analyses could be significantly flawed. Harbig et al. [2005] re-annotated the Affymetrix U133 plus 2.0 arrays using BLAST matching against documented and postulated human transcripts. They redefined ∼37% of the probes and identified more than 5,000 probesets that detected multiple transcripts. In addition to ensuring that probes detect the correct gene, with improvements in annotation and subsequent probe refinement, fewer probes on commercial arrays will hybridize to multiple splice variants, show cross-hybridization to other genes in the same family and hybridize to nonspecific probes. Therefore, as a result of errors in annotation, early studies that matched genes based on the annotation provided by the manufacturer, or by the cDNA clone set provider, were examining a large portion of incorrectly matched gene sets.

Errors in annotation continue to be an issue that affects every microarray technology. However, major improvements have been made as more sequence information is curated, validated, and annotated in high-quality databases such as Refseq [http://www.ncbi.nlm.nih.gov/RefSeq/]. In addition, in early studies probe sequence was not available and users had to trust manufacturer gene identification. Today, a large portion of microarray platform providers make all probe sequence information available; in addition, MIAME guidelines require submission of probe sequences for each spot on a microarray [http://www. mged.org/Workgroups/MIAME/miame.html]. Cross-checking probe sequence annotation is an important first step for validation of expression changes for any gene.

Other platform issues that relate to potential discrepancies in cross-platform comparisons result from sub-optimal printing, labeling, hybridizing, and washing methods in early studies [Kuo et al., 2006]. In general, several of the early studies suffered from lack of technical expertise with microarrays and more specifically, with one of the platforms in their comparison. Poor quality data will be generated when inexperienced technicians carry out the hybridization and/or sub-optimal protocols are used. The realization and control of the effect of environmental influences, such as ozone [Fare et al., 2003] on the fluorescent chemicals used, have also resulted in improved acquisition of data. Finally, the general quality of printing of both cDNA and oligonucleotide microarrays has improved significantly over the past 5 years.

In summary, methodological and platform improvements have been made over the past several years that have resulted in a decrease in the observed technical variability and resulted in superior performance. The result has been a general increase in the measured correlation among DNA microarray technologies.

## Experimental Design and Analysis Issues

Many of the studies described in Table I suffer from flaws in experimental design. In some studies, data were generated in different laboratories at different times using different samples [Kuo et al., 2002]. To directly evaluate the correlation among technologies, the exact same RNA sample should be used across all experiments [Kawasaki, 2006]. Biological variability and tissue heterogeneity will significantly contribute to variance between the datasets. In addition, many of the early studies did not apply a large enough sample size, including both technical and biological replicates, to arrive at the conclusions drawn. Lastly, to investigate differential expression, samples that are sufficiently distinct should be examined [Kuo et al., 2006].

An important pre-processing step involves filtering microarray image data for poor quality, saturated, and low-signal spots. Poor quality spots and saturated signal do not accurately represent the expression of a gene. As discussed earlier, signals near background and reaching saturation do not provide accurate or reliable measures of gene expression. Stringent filtering methods were not routinely applied to data in early microarray studies and would have greatly improved the quality of these datasets.

Appropriate statistical tools, including normalization, clustering, and identification of differentially expressed genes need to be applied in any microarray experiment. Microarray normalizations and statistical analyses have changed over time and current methods are superior to those applied in the early studies. In addition, microarrays should be normalized both within and between the technologies, incorporating a normalization approach across all of the data in the experiment [Kuo et al., 2006]. A major finding of the MAQC consortium was that the correct tools need to be applied to identify differentially expressed genes [Guo et al., 2006]. Surprisingly, the authors found that traditional parametric analyses and other microarray-tailored analyses may not derive comparable gene lists from alternative technologies. The authors suggest that gene lists generated by fold-change ranking were more reproducible than those using other methods. More work needs to be done to determine the most accurate and reproducible methods for deriving lists of differentially expressed genes from different technologies.

Early studies examining overall expression level of genes ignored the influence of probe position and sequence on the derived signal intensity [Draghici et al., 2006]. Improved correlations were generated when relative ratios were compared rather than absolute measures of gene expression in the later studies. Therefore, the measurement of ratios (to a control or reference sample) rather than signal intensities is now generally applied in cross-platform analyses. In addition, microarrays do not provide quantitative measures, and are therefore not very precise or accurate. As a result, absolute magnitude of a change should not be compared across platforms. Rather, emphasis should be placed on the direction of change [Kawasaki, 2006].

All of the above factors contributed to the discrepancies observed in the first studies examining the correlation of expression profiles across DNA microarray technologies. Subsequent changes in methods yielded improved correlation metrics, as described in the remaining sections.

## CONCLUSIONS

In general, microarray platforms and associated technologies and tools have improved greatly over the past decade. As potential sources of error and reasons for discrepancies between technologies are uncovered, the relationship among gene expression data produced using the different platforms is becoming more clear. Some key points include: (a) probe sequence will affect measured intensity; (b) relative ratios are more comparable than absolute measures; (c) annotation problems still complicate analysis and genes should be evaluated at the sequence level; (d) stringent filtering leads to more reproducible and comparable measurement of gene expression; (e) normalization and method of data analysis will affect the derived gene expression profiles; (f) validation using an alternative method is required. The laboratory and platform effect remains a major issue and comparisons need to be drawn carefully. Ensuring the appropriate experimental design before making comparisons between datasets is critical to acquiring meaningful correlations. Several of the above points (in particular (d), (e), and (f)) are good microarray practice in general, and should apply to any experiments employing this technology.

The development of standardized protocols for everything from RNA labeling to data handling will also improve the measured correlation between platforms and laboratories. Increased automation will lead to lower technical variability and result in higher correlation among technologies [Yauk et al., 2005]. The development of internal and external controls will facilitate evaluation of data quality [van Bakel and Holstege, 2004; Yauk et al., 2006]. Implementation of standards and references will lead to a better understanding of the relationship among the gene expression measures from different technologies [Andersen and Foy, 2005; Kawasaki, 2006]. Decreasing intra-platform variability is an important first step towards ensuring that microarrays produce robust and reproducible data.

In conclusion, the vast majority of papers published over the past several years support a high degree of correlation among microarray technologies. Evaluation of gene expression using alternative approaches (e.g. quantitative real-time PCR) also supports the conclusion that microarrays provide reliable and reproducible measures of transcript levels and profiles. When data are acquired and handled correctly measures of gene expression are highly correlated. This review provides a framework identifying several key features of general good microarray practice, as well as identifying critical mechanisms to ensure that data produced by different microarray technologies are comparable.
